# Age-Related Physical Activity Determinants in Breast Cancer Survivors: A Theory-Based Multigroup Analysis

**DOI:** 10.1093/geroni/igaf122.3212

**Published:** 2025-12-31

**Authors:** Jeongeun Hwang, Xirong Cui, Ah Rim Lee, Moonkyoung Park, Eunyoung Park, Hyun-E Yeom, Misook Jung

**Affiliations:** Chungnam National University, Daejeon, Republic of Korea; Chungnam National University, Daejeon, Republic of Korea; Chungnam National University, Daejeon, Republic of Korea; Chungnam National University, Daejeon, Republic of Korea; Chungnam National University, Daejeon, Republic of Korea; Chungnam National University, Daejeon, Republic of Korea; Chungnam National University, Daejeon, Republic of Korea

## Abstract

**Background:**

Physical activity is a proven non-pharmacological intervention that alleviates treatment-related symptoms, enhances cardiovascular health, lowers cancer recurrence and mortality risks, and improve daily functioning and quality of life. However, older breast cancer survivors tend to be less active than younger survivors. This age-related disparity may stem from challenges unique to older survivors-such as reduced self-efficacy due to physical limitations or past negative experiences-which can diminish their motivation to initiate or maintain exercise routines. Yet, it remains unclear whether these motivational factors influence physical activity engagement differently across age groups. This study compared the effects of motivational and volitional factors on physical activity among older and younger cancer survivors.

**Methods:**

A total of 218 older and 287 younger breast cancer survivors were recruited. Key constructs from the Health Action Process Approach-including self-efficacy, planning, risk perception, outcome expectancy, intention, and physical activity-were assessed. Structural equation modeling and multigroup analysis were employed to examine the data.

**Results:**

Motivational constructs explained 27.8% of the variance in intention among older survivors compared to 3.2% among younger survivors, while volitional constructs explained 42.1% of physical activity variance for older survivors and 88.1% for younger survivors. Significant group differences were observed in the pathways linking risk perception to intention, action planning to physical activity, and recovery self-efficacy to physical activity.

**Conclusion:**

These findings indicate that motivational factors exert stronger influences on older survivors, whereas volitional factors are more critical for younger survivors. Tailored interventions addressing these age-specific influences may be key to enhancing physical activity engagement.

